# Physicochemical Quality, Polyphenol Profiles, and Postharvest Performance of Florida Pearl^®^ ‘FL 16.78-109’ White Strawberries Compared to the Red Cultivar ‘Florida Brilliance’ †

**DOI:** 10.3390/foods12173143

**Published:** 2023-08-22

**Authors:** Alyssa Nicole Smith, Maria Cecilia do Nascimento Nunes

**Affiliations:** Food Quality Laboratory, Department of Cell Biology, Microbiology and Molecular Biology, University of South Florida, Tampa, FL 33620, USA

**Keywords:** *Fragaria chiloensis* spp. *chiloensis* f. *chiloensis*, *Fragaria* × *ananassa* Duch., weather conditions, harvest date, cold storage, color, firmness, sugars, ascorbic acid

## Abstract

White-fruited strawberry cultivars have recently become popular due to their exotic appearance and flavor, but more needs to be known about their overall quality and postharvest performance. The objective of this study was to characterize and compare the overall quality of the white-fruited strawberry Florida Pearl^®^ ‘FL 16.78-109’ against the commercial, red-fruited strawberry ‘Florida Brilliance’ at harvest and during cold storage (1 °C). Results showed that harvest date and weather conditions contributed to significant differences in fruit quality, regardless of the cultivar. However, Pearl was softer at harvest and had lower total phenolic and anthocyanin contents but was less acidic and had higher total sugars and ascorbic acid contents than Brilliance. Pearl major polyphenols were kaempferol 3-glucoside, quercetin 3-glucoside, quercetin, and gallic acid, while for Brilliance epicatechin, pelargonidin, pelargonidin 3-glucoside, and ferulic acid were the major polyphenol compounds identified. After cold storage, Pearl lost less weight than Brilliance and showed a less dramatic decline in individual polyphenols. Pearl and Brilliance anthocyanins and phenolic acids were the polyphenol groups most affected by cold storage because they showed the highest decline from harvest to the end of storage. Cold storage also had different effects on other polyphenols, but the effect was cultivar-dependent. Overall, white strawberries have a unique appearance, are sweet, have an excellent bioactive profile, and can maintain good postharvest quality.

## 1. Introduction

Florida is the winter strawberry capital of the United States, and while the traditional red strawberries are always popular due to their characteristic color and flavor, the white-fruited strawberry is becoming trendy. The white Chilean strawberry *Fragaria chiloensis* spp. *chiloensis* f. *chiloensis* is a native of southern Chile and one of the progenitors of the common red strawberry *Fragaria* × *ananassa* Duch. [1,2]. Depending on the cultivation practices (i.e., open fields or tunnels with UV-filtering plastic), the cultivated *F. chiloensis* presents a wide range of surface colors from almost white to slightly red [1]. In Chile, white strawberries are still cultivated and have, in recent years, become trendier in other parts of the world [1,3]. Because of their tropical fruit and pineapple-like aroma [4], white strawberries were initially called “Pineberries” and were very appreciated in Europe in the 19th century. In recent years, white strawberry cultivars have become a gourmet fruit, and in Florida, the white strawberries Florida Pearl^®^ ‘FL 16.78-109’ reached the market around 2020 [5]. However, little is known about the postharvest handling of white-fruited Chilean strawberries [3], and so far, no data has been published on the biochemical characteristics and postharvest performance of the white Florida Pearl^®^ ‘FL 16.78-109’ strawberry.

Like almost all living organisms, the strawberry fruit has an internal mechanism that protects it from UV radiation. Anthocyanins are the polyphenolic compounds responsible for the red color of strawberry fruit and are significant contributors to its total phenolic content [6]. The development of red pigmentation occurs when anthocyanins are synthesized in response to UV-light exposure, acting as a screen to minimize photo-oxidative damage to photosynthetic tissues [7,8,9,10,11]. This protection mechanism can be seen as a deepening of the strawberry fruit’s overall red coloring as the UV index increases. White strawberries do not develop the typical red color because they have significantly lesser amounts of anthocyanins due to several enzymes in the biosynthetic anthocyanin pathway being downregulated [12,13,14]. The downregulated enzymes chalcone synthase, dihydroflavonol reductase, flavanone 3-hydroxylase, and methyltransferase belong to the flavonoid biosynthetic pathway, which produces several compounds, including pelargonidin, that gives the red color to strawberry fruit [12]. Earlier studies showed that, while total anthocyanin contents are much higher in red strawberries, white *Fragaria chiloensis* strawberries have higher levels of ellagic acid [15,16,17]. Unlike in the red-fruited strawberry varieties, where pelargonidin 3-glucoside is the major anthocyanin, in white-fruited strawberries, cyanidin 3-glucoside predominates primarily because of the achenes being the most colored tissues in the whole fruit [16,18,19]. This lesser concentration of natural UV protective compounds can lead to the hot Florida weather impacting white-fruited strawberries more than their red-fruited counterparts. Elevated field temperatures and UV radiation may also significantly impact other major strawberry metabolites. It has been previously reported that pre- and postharvest temperatures can affect the fruit’s metabolism, resulting in higher respiration rates [20,21]. Exposure of strawberries to increasing environmental temperatures contributes to fruit softening and decreases primary and secondary fruit metabolites [22,23].

This study aimed to characterize and compare the overall quality of the white-fruited strawberry Florida Pearl^®^ ‘FL 16.78-109’ against the commercial, red-fruited strawberry ‘Florida Brilliance’ at harvest and postharvest. To accomplish this objective, the two strawberry cultivars were harvested simultaneously in January, February, and March, during the 2021 strawberry season. Analytical color and texture, weight loss, total and individual sugars, ascorbic acid, phenolic and anthocyanin contents, and individual polyphenols were measured at harvest and after nine days at 1 °C. This is the first study characterizing and comparing the physicochemical quality and polyphenol profiles of the white-fruited Florida Pearl^®^ ‘FL 16.78-109’ strawberry against the commercial red strawberry cultivar ‘Florida Brilliance’ at harvest and after cold storage.

## 2. Materials and Methods

### 2.1. Plant Material

The two strawberry cultivars used in this study were the white Florida Pearl^®^ ‘FL 16.78-109’ and red ‘Florida Brilliance’ (hereafter referred to as Pearl and Brilliance, respectively). Both cultivars were harvested from experimental fields at the University of Florida Gulf Coast Research and Education Center in Wimauma, Florida, USA (27.76° N; 82.23° N; 39.95 m). Strawberries were harvested during the 2021 season on 18 January, 10 February, and 1 March. Two flats of Brilliance containing ≈300 strawberries each and one flat of Pearl containing ≈120 strawberries were harvested in January. Two flats of Brilliance and Pearl, each containing ≈300 strawberries, were harvested in February and March. The fruit was transported to the University of South Florida Food Quality Laboratory in Tampa within one hour of harvest under air-conditioning and sorted into commercial clamshells (capacity ≈ 0.453 kg). The experiment was repeated three times (January, February, and March), and the experimental set-up was the following: for non-destructive analysis (i.e., color and weight loss), three replicated clamshells containing 15 fruit each were used for day 0, and the same clamshells were also used for the non-destructive evaluations on day 9. For destructive evaluations (i.e., texture, moisture content, pH, acidity, soluble solids, sugars, ascorbic acid, phenolics, anthocyanins, and polyphenols), three clamshells containing 15 fruit each were used on day 0, and the same number on day 9 (i.e., a total of nine clamshells containing 15 fruits each were used per experiment/harvest per cultivar).

### 2.2. Weather Conditions

Weather conditions at harvest (18 January, 10 February, and 1 March 2021) were retrieved from the Florida Automated Weather Network [24].

### 2.3. Storage Conditions

As reported previously, optimum storage conditions for strawberries are temperatures close to 1 °C with high relative humidity (RH) [25]. Therefore, strawberry samples were stored at 1 ± 0.2 °C and 85.0 ± 0.5% RH (VPD = 0.23 KPa) inside temperature- and RH-controlled chambers (Forma Environmental Chambers Model 3940 series, Thermo Electron Corporation, Cincinnati, OH, USA). The experiments were terminated after 9 days of storage when the fruit started to show signs of decay. The temperature and relative humidity (RH) were monitored throughout the study using HOBO^®^ brand U12 data loggers (Onset Computer Corporation, Pocasset, MA, USA), which record within an accuracy of ±0.35 °C. The RH was monitored using HOBO^®^ brand U12 data loggers (Onset Computer Corporation, Pocasset, MA, USA), which record within an accuracy of ±2.5% from 10 to 90% RH.

### 2.4. Instrumental Color and Texture Analysis

Two-color measurements were taken on opposite sides of the fruit in the equatorial region. A hand-held tristimulus reflectance colorimeter (Model CR-400, Minolta Co., Ltd., Osaka, Japan) was used following the procedure described by Kelly et al. [26]. The firmness of each strawberry was measured using a TA.XT Plus Texture Analyzer (Texture Technologies Corp., Brewster, NY, USA) as described by Whitaker et al. [27]. The force required to compress the fruit by 3 mm was recorded in kgf and converted to newtons (N = kgf × 9.8).

### 2.5. Weight Loss and Dry Weight

Weight loss of three replicate samples of 15 strawberries was calculated from the fruit’s initial weight and after 9 days. Concentrations of chemical constituents at harvest (day 0) were expressed in fresh and dry weight. After storage (day 9), they were expressed in dry weight to show the differences between strawberry cultivars that might be obscured by differences in the water content [28].

### 2.6. Acidity and Soluble Solids Content

Three replicate samples of 15 individual fruit per treatment were homogenized in a laboratory blender at high speed for 2 min. The resulting puree was immediately frozen and kept at −30 °C until used. Titratable acidity (TA) and soluble solids content (SSC) were determined according to Nunes et al. [29].

### 2.7. Sugar Analysis

Frozen samples were thawed at 4 °C overnight, and 2 g of strawberry fruit puree (from each of three replicate samples of 15 strawberries each) was combined with 8 mL of ultrapure water (Ω18–17) and then centrifuged at 1811× *g* for 10 min. The obtained supernatant was filtered through a 0.45 µm nylon filter into 0.002 L labeled vials. Quantification of sucrose, fructose, and glucose was conducted using a Hitachi HPLC with a refractive index detector and a 300 mm × 8 mm Shodex SP0810 column (Shodex, Colorado Springs, CO, USA) with an SP-G guard column (2 mm × 4 mm) as described by Kelly et al. [26].

### 2.8. Ascorbic Acid Analysis

Total ascorbic acid (AA) was quantified by mixing 2 g of strawberry fruit puree (from each of three replicate samples of 15 strawberries) with 20 mL metaphosphoric acid mixture (6% HPO_3_ containing 2 N acetic acid). Samples were then filtered (0.22 μm) before HPLC analysis. The ascorbic acid analysis was conducted using a Hitachi LaChromUltra UHPLC system with a diode array detector and a LaChromUltra C18 2 μm column (2 × 50 mm) (Hitachi, Ltd., Tokyo, Japan) as described by Kelly et al. [26].

### 2.9. Total Phenolics and Anthocyanin Analysis

Total phenolic compounds (TPC) were measured using the Folin–Ciocalteu reagent following the procedure described by Nunes et al. [30]. Anthocyanins (ANC) were extracted in 0.5% (*v*/*v*) HCl in methanol and measured using the procedure described by Nunes et al. [30].

### 2.10. Polyphenol Extraction

The extraction of the polyphenols was performed as described by Abountiolas et al. [31] with minor adjustments, such as using the fruit’s puree instead of juice. Triplicates of 5 g of homogenate were mixed with 15 mL of acetone and homogenized using a polytron for 1 min. Samples were sonicated for 10 min, and finally filtered through Whatman paper No. 4. The filtrate was concentrated to 5 mL in an SPD121P SpeedVac^®^ Concentrator (Thermo Fisher Scientific Inc., Asheville, NC, USA) and finally passed through a classic C18 Sep-Pack cartridge (Waters Technologies Corp., Milford, MA, USA). Before passing the concentrated sample, the Sep-Pack cartridge was activated with ~5 mL of methanol, followed by ultrapure water and 3% acidified water. Anthocyanins and other phenolic compounds were absorbed into the cartridge, while sugars, acids, and other water-soluble compounds were eluted with ~10 mL of acidified water. The phenolic compounds were then recovered by passing ~2 mL of methanol (containing 3% formic acid) through the cartridge. The extracted sample was filtered through a 0.20 µm syringe filter into 2 mL autosampler vials and stored at −30 °C until analysis.

### 2.11. Individual Polyphenol Analysis

Analysis of phenolic compounds was conducted using a Hitachi LaChroma Ultra HPLC system coupled with a photodiode array detector (Hitachi, Tokyo, Japan), according to Abountiolas et al. [31], with minor adjustments regarding retention times and dry weight conversions. Samples were injected at 40 °C into a reverse-phase Hypersil Gold C18 column (100 × 2.1 mm; particle size, 1.9 µm) (Thermo Fisher Scientific Inc., Waltham, MA, USA). The two mobile phases consisted of acidified water containing 0.5% formic acid (mobile phase A) and 0.1% formic acid in acetonitrile (mobile phase B) in an isocratic mixture. The flow rate was 0.3 µL/min, the wavelength detection included 250, 280, 360, and 520 nm, and the sample injection volume was 10 µL. Retention times and spectra were compared with pure standards of 17 compounds (at concentrations of 0.005 mg/mL, 0.01 mg/mL, and 0.1 mg/mL) from different polyphenol classes: flavonoids (cyanidin, pelargonidin, cyanidin 3-glucoside, pelargonidin 3-glucoside, quercetin, kaempferol, quercetin 3-glucoside, kaempferol 3-glucoside, myricetin, catechin, and epicatechin), phenolic acids (*p*-coumaric acid, ferulic acid, caffeic acid, gallic acid, and chlorogenic acid), and hydrolyzable tannins (ellagic acid). The flavonoids can be further classified into anthocyanidins (cyanidin and pelargonidin), anthocyanins (cyanidin 3-glucoside, and pelargonidin 3-glucoside), flavonols (quercetin, kaempferol, quercetin 3-glucoside, kaempferol 3-glucoside, and myricetin), and flavanols (catechin and epicatechin).

### 2.12. Statistical Analysis

The Statistical Analysis System computer package (SAS Institute, Inc., SAT 15.1. 2018, Tokyo, Japan) was used to analyze the data from these experiments. The data were analyzed via three-way ANOVA with harvest, cultivar, and storage time as the independent variables. Statistical analysis showed a significant difference between harvests leading to separate analyses for each harvest. Significant differences between treatments were detected using Tukey’s Studentized Range (HSD) test at the 5% significance level.

## 3. Results and Discussion

The physicochemical characteristics and polyphenol profiles of the white and red-fruited strawberry cultivars Pearl and Brilliance were measured and compared at harvest and after cold storage. Note that, to compensate for water loss during storage, chemical compounds are expressed in mg 100 g^−1^ on a dry weight (DW) basis, except individual and total sugars, which appear expressed in g 100 g^−1^ because they are generally present in the fruit in higher amounts than the remaining chemical compounds. Average values for chemical compounds obtained for day 0 (at harvest) are also presented on a fresh weight (FW) basis. This allows data from our study to be compared to previously published values, often presented on a fresh weight basis.

### 3.1. Color: Differences between Pearl and Brilliance, Seasonal Variability, and Impact of Storage

**Figure 1:**
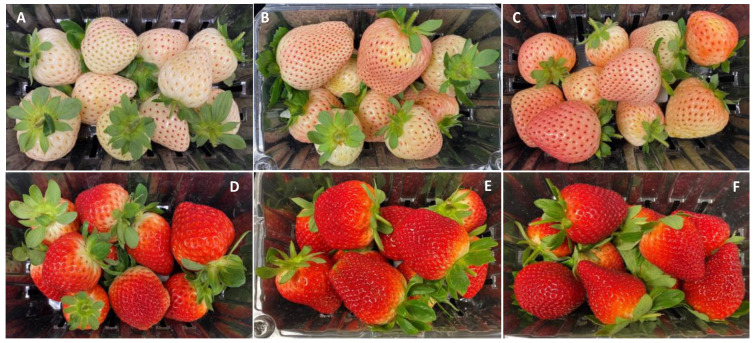
Appearance of white-fruited Pearl and red-fruited Brilliance strawberry cultivars on the day of harvest (day 0). (**A**,**D**) January harvest; (**B**,**E**) February harvest; (**C**,**F**) March harvest.

**Figure 2:**
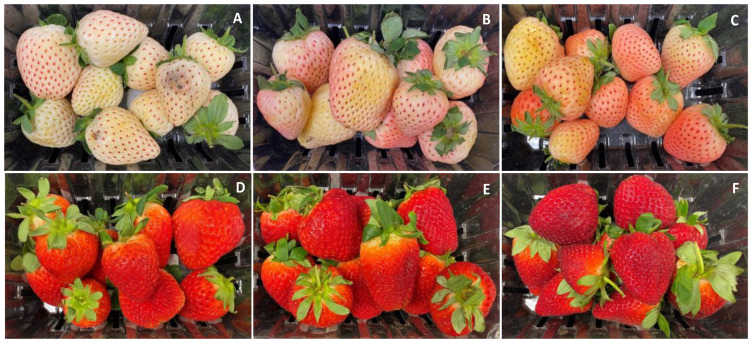
Appearance of white-fruited Pearl and red-fruited Brilliance strawberry cultivars after 9 days at 1 °C. (**A**,**D**) January harvest; (**B**,**E**) February harvest; (**C**,**F**) March harvest.

There was a noticeable variability in Pearl’s surface color as the season progressed and clear changes occurred in the appearance of the fruit after 9 days of cold storage (Figure 1 and Figure 2). In January, the color of Pearl strawberries appeared, at harvest, less pinkish and rather whitish-cream with small reddish achenes (Figure 1A) compared to the fruit harvested later in the season, which had a noticeable pinkish-red blush (Figure 1B,C). After storage, Pearl strawberries lost their creamy appearance, developing a yellowish discoloration, with fruit harvested in January also showing signs of decay (Figure 2A–C). As the season progressed, Brilliance strawberries showed visible color changes from a brighter light to a darker red (Figure 1D–F). After storage, the red coloration increased, and the fruit appeared less glossy than at harvest (Figure 2D–F).

**Figure 3:**
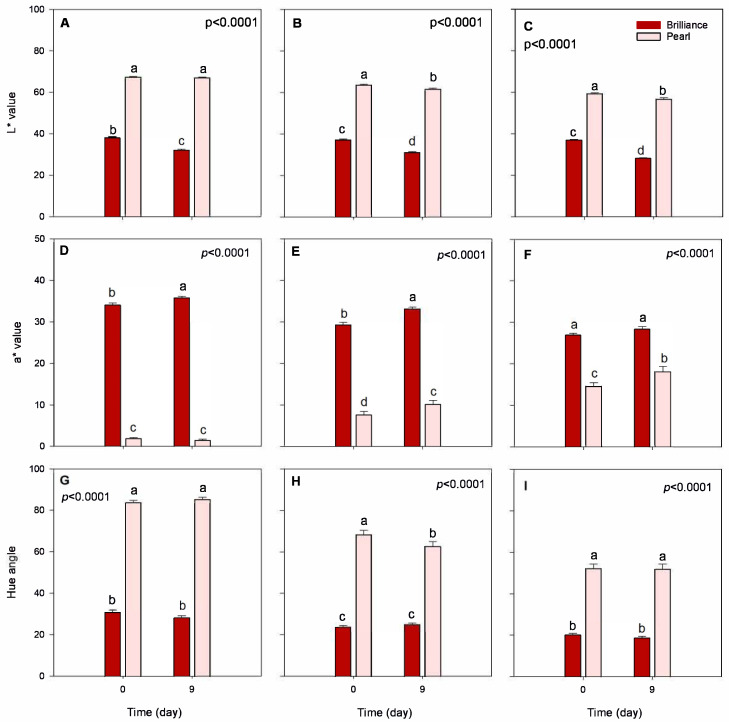
Surface color, lightness (L*), redness (a*), and hue of Brilliance and Pearl strawberry cultivars at harvest (day 0) and after 9 days at 1 °C. (**A**,**D**,**G**) January harvest; (**B**,**E**,**H**) February harvest; (**C**,**F**,**I**) March harvest. Bars are means of 3 biological replicates of 15 strawberries each. The letters above each bar denote significant differences (*p* < 0.05) between cultivars and days based on Tukey’s HSD test; bars with the same letter are not significantly different.

Data from instrumental color analysis confirmed the subjective appearance observations for both strawberry cultivars (Figure 3). Overall, Pearl had significantly higher L* (lighter) and hue angle (less red) values at harvest and after storage than Brilliance. These results were expected, as Brilliance shows a moderate external redness [32]. For a* value, Brilliance had significantly higher values (redder) than Pearl for all three harvest dates, both at harvest (a* = 30.1 for Brilliance and a* = 8.0 for Pearl) and after storage (a* = 32.4 for Brilliance and a* = 10.1 for Pearl). These a* values agree with those previously published for Pearl and Brilliance [5,32]. Previous studies have also reported that increased field temperatures during growth contributed to decreased L* and hue angle values because the strawberry surface and the flesh become darker, redder, and more intensely pigmented [22,23].

Anthocyanins are the primary pigment contributing to the red color of strawberries, and it has been reported that exposure to UV rays can promote their synthesis [9,11]. The average field temperature was higher during the February and March harvests than in January, and the average UV index was also greater (Table 1). The increase in UV index as the season progressed, along with the stress of the field heat, likely contributed to an increase in anthocyanin synthesis and enhancement of red coloring of Pearl strawberries [5]. Anthocyanins seem to play a major role in protecting strawberry fruit from UV radiation [7,8], and since Pearl strawberries lack a significant amount of these protective compounds, as the UV index increased, the production of anthocyanins needed to increase to allow the fruit to grow fully. The effect of UV radiation on the increased redness of strawberry Pearl was also confirmed, as shown below, by the significant increase in total anthocyanin content over the season.

After 9 days of cold storage, there was a significant decrease in the L* and an increase in the a* values of Pearl and Brilliance, particularly in the later harvests, reflecting the development of a darker appearance (Figure 2 and Figure 3). A previous study also showed that L* value of white-fruited Chilean strawberries significantly decreased after 4 days at 20 °C, whereas there was no significant difference in the a* values throughout storage [33].

### 3.2. Firmness: Differences between Pearl and Brilliance, Seasonal Variability, and Impact of Storage

**Figure 4:**
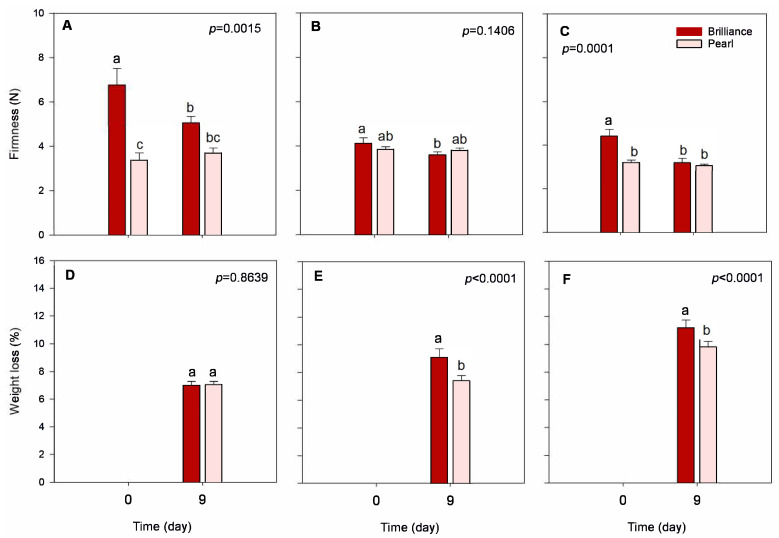
Firmness and weight loss of Brilliance and Pearl strawberry cultivars at harvest (day 0) and after 9 days at 1 °C. (**A**,**D**) January harvest; (**B**,**E**) February harvest; (**C**,**F**) March harvest. Bars are means of 3 biological replicates of 15 strawberries each. The letters above each bar denote significant differences (*p* < 0.05) between cultivars and days based on Tukey’s HSD test; bars with the same letter are not significantly different.

Brilliance was significantly firmer at harvest than Pearl, particularly fruit harvested in January (Figure 4A–C). The white strawberry *F. chiloensis* has been reported as being softer and softening faster during ripening than the commercial red cultivar ‘Chandler’ due to accelerated enzyme-mediated degradation of the cell walls [34,35,36]. This notwithstanding, the content of water-soluble pectin in *F. chiloensis* compared to the cultivar ‘Chandler’ seems to be a determinant of its softer cortical tissue [37]. In our study, Pearl and Brilliance firmness at harvest was, on average, 3.5 N and 5.1 N, respectively. Others have, however, reported that during ripening *F. chiloensis* softens faster than the cultivar ‘Chandler’, but that at the ripe stage, their firmness is similar—3.36 N and 3.15 N, respectively [35,36]. These are values comparable to the ones we obtained for Pearl but lower than the firmness obtained for Brilliance. In previous studies, we reported higher firmness values for ‘Chandler’ (between 7.0 and 9.0 N) [38,39] than those reported by Figueroa et al. [35,36] but lower than those reported by Singh et al. (1.3 N) [40]. Also, higher firmness values than those obtained in our study for Pearl were previously reported for the white-fruited strawberry *F. chiloensis* [41]. Thus, while it seems somehow necessary to compare firmness values for the same strawberry cultivar obtained from different sources, one should also consider that the variability in the data might come from the differences in, for example, weather conditions, growing areas, agricultural practices, and analytical procedures.

Strawberries harvested in January were firmer than those from later harvests, regardless of the cultivar (Figure 4A–C). The decrease in fruit firmness as the season progressed might be related to increased field temperatures observed from January to March (Table 1). In a previous study, Kim et al. [23] also reported that the firmness of strawberry fruit decreases, regardless of the cultivar, as the temperature during growth increases. Whitaker et al. [32] also reported a slight decrease in the firmness of Brilliance as the season progressed.

Both strawberry cultivars softened during cold storage, but after 9 days, the difference in firmness between cultivars was not significantly different. Compared to Brilliance, Pearl strawberries maintained their firmness better during cold storage. For example, after cold storage, the firmness of Brilliance harvested in March decreased by 27.6%, while the firmness of Pearl decreased by only 4.4%. According to Moya-León et al. [34], fruit that softens faster generally has a shorter shelf life and is more susceptible to fungal decay than firmer fruit. Thus, even though Brilliance was firmer at harvest, it softened faster during storage than Pearl. A previous study showed that the firmness of white-fruited Chilean strawberries significantly decreased after 4 days at 20 °C [33]. Saavedra et al. [41] also reported a decrease in the firmness of *F. chiloensis* during storage for 72 h at 22 °C. And another study showed that the firmness of Chilean strawberries *F. chiloensis* stored for 8 days at 2 °C plus 48 h at 20 °C decreased from 1.42 N at harvest to 1.01 N after storage [42], corresponding to a 28.9% decrease. In our study, the lower decrease in Pearl firmness during storage was likely because we used colder storage conditions than Figueroa et al. [42].

### 3.3. Chemical Composition: Differences between Pearl and Brilliance, Seasonal Variability, and Impact of Storage

#### 3.3.1. Weight Loss

As expected, weight loss increased during storage, regardless of the cultivar or harvest date (Figure 4D–F). However, Pearl harvested in February and March had a significantly lower weight loss than Brilliance. Differences in morphological characteristics between strawberry genotypes (e.g., the thickness of the cuticle and achene and stomata density) may contribute to differences in weight loss between cultivars [43]. In a previous study, Figueroa et al. [42] reported that the weight of white-fruited Chilean strawberries *F. chiloensis* stored for 8 days at 2 °C plus 48 h at 20 °C decreased from 13.69 g at harvest to 10.01 g after storage, which corresponds to a 26.8% decrease. Although the negative impact of postharvest temperature on strawberry quality has been well documented, preharvest field temperatures may also affect the quality of the fruit. In our study, weight loss increased as the season progressed, with Brilliance and Pearl harvested in March having the highest weight loss (11.2% and 9.8%, respectively) when compared to the fruit harvest in January (7.0% and 7.1%, respectively) or February (9.1% and 7.4%, respectively). The higher field temperatures measured in March (Table 1) than in January and February could have contributed to a more significant water loss during storage. Barrios et al. [20] showed that the respiration rate of strawberries grown at higher temperatures (23 °C compared to 10 °C) significantly increases due to accelerated metabolic reactions. As shown in Table 1, by March, a significant increase in the field temperature could have been an important factor contributing to the increased postharvest weight loss observed throughout the season and possibly greater respiration rate.

#### 3.3.2. Acidity and Soluble Solids Content

**Figure 5:**
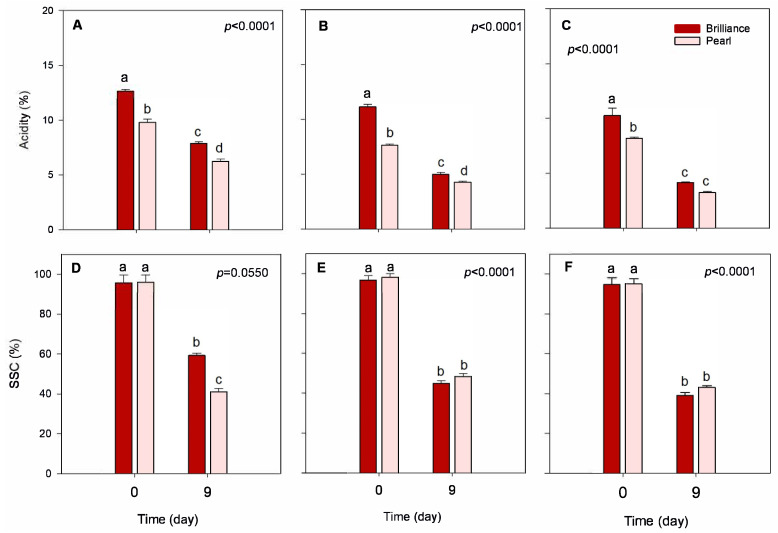
Acidity and soluble solids content (SSC) of Brilliance and Pearl strawberry cultivars at harvest (day 0) and after 9 days at 1 °C. (**A**,**D**) January harvest; (**B**,**E**) February harvest; (**C**,**F**) March harvest. Bars are means of 3 biological replicates of 15 strawberries each. The letters above each bar denote significant differences (*p* < 0.05) between cultivars and days based on Tukey’s HSD test; bars with the same letter are not significantly different.

Acidity (TA) levels remained stable during the season regardless of the cultivar, showing only a slight decrease as the season progressed (Figure 5A–C). In a previous study, Palmieri et al. [10] reported that, in general, strawberry cultivars grown in warmer locations and exposed to higher levels of UV radiation have lower acidity than those grown under lower temperatures and UV radiation. Wang et al. [22] also reported that higher field temperatures during strawberry growth reduce fruit acidity and soluble solids content (SSC), with plants growing at lower night and day temperatures producing, in general, fruit with higher TA and SSC than those from higher temperatures. However, in our study, the effect of harvest date on the TA or SSC was very subtle. At harvest, Pearl strawberries were, on average, significantly less acidic than Brilliance (8.39% and 11.36% acidity on a DW basis, respectively) (Table 2). Whitaker et al. [5] suggested that the low-acid flavor profile of Pearl is very distinct, which makes it non-comparable to the traditional red strawberries. On an FW basis, at harvest, Brilliance and Pearl had, on average, 0.78% and 0.65% acidity (Table 2), which are values comparable to those published previously [5,32]. After storage, the TA of both cultivars tended to decrease, regardless of the harvest, with differences between cultivars becoming less pronounced. For example, after 9 days of storage, there was no significant difference in the TA of Brilliance and Pearl harvested in March (Figure 5C). However, Molinett et al. [33] reported that the TA of white Chilean strawberries increased after 6 days at 20 °C. Compared to our study, the increase in the TA values reported by Molinett et al. [33] might have resulted from the concentration effect caused by weight loss during storage which, unlike in our study, was not considered.

At harvest, there was no significant difference between the SSC of Brilliance and Pearl (Figure 5D–F). However, on average, Pearl tended to have slightly higher SSC than Brilliance (Table 2). Similar SSC values to those obtained in our study were previously reported for Brilliance and Pearl harvested over several seasons. Overall, at harvest, the SSC of Brilliance was either lower than or like that of Pearl [5]. There was no significant variability in the SSC of both cultivars over the season. However, Brilliance and Pearl harvested in March had slightly lower SSC (94.72% and 95.08%, respectively) than fruit harvested in February (96.77% and 98.17%, respectively). In a previous study, though, Palmieri et al. [10] reported that, in general, strawberry cultivars grown in warmer locations and exposed to higher levels of UV radiation had higher SSC than those grown under lower temperatures and UV radiation.

After storage, SSC decreased significantly, regardless of the cultivar (Figure 5). The decrease in SSC of Brilliance and Pearl was higher in fruit harvested in February (53.5% and 50.8% decrease, respectively) and March (58.7% and 54.50% decrease, respectively) than in January (38.0% and 32.7% decrease, respectively). In a previous study, Molinett et al. [33] also showed that SSC significantly decreased in white Chilean strawberries stored for 6 days at 20 °C. Also, Figueroa et al. [42] reported that the SSC of white Chilean strawberries *F. chiloensis* stored for 8 days at 2 °C plus 48 h at 20 °C decreased from 9.55 g 100 g^−1^ FW at harvest to 8.80 g 100 g^−1^ FW after storage, which corresponds to a 7.9% decrease. Again, compared to our study, the smaller decline in the SSC reported by Figueroa et al. [42] might have resulted from the concentration effect caused by weight loss during storage which, unlike in our study, was not considered.

#### 3.3.3. Sugar Profiles

**Figure 6:**
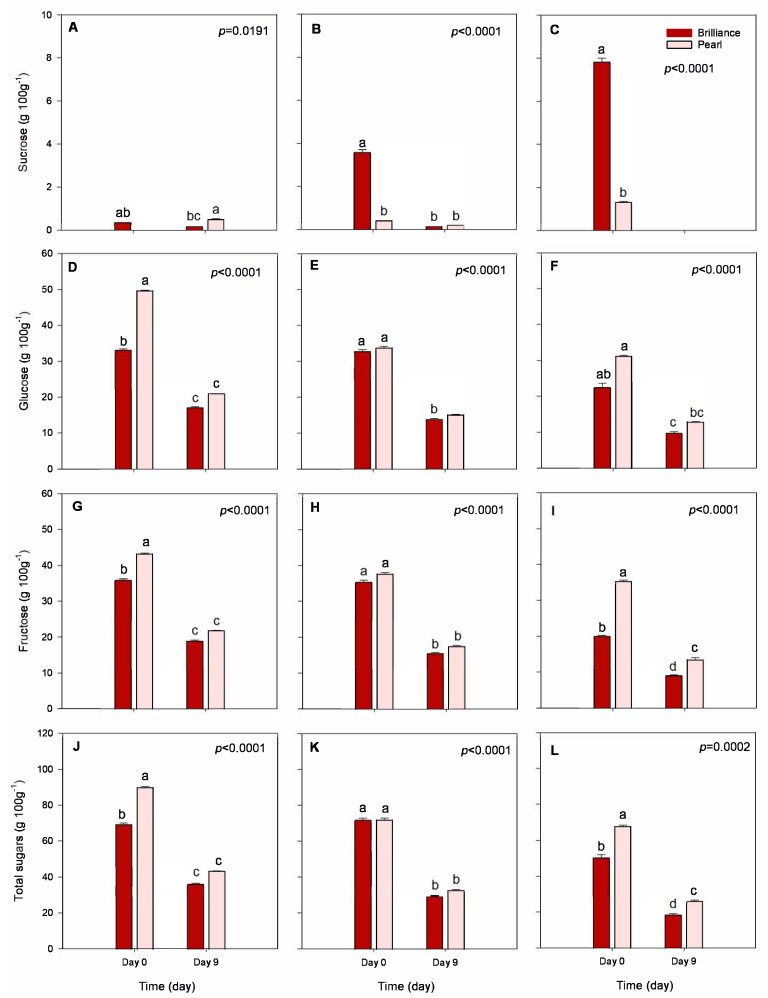
Sucrose, glucose, fructose, and total sugar contents of Brilliance and Pearl strawberry cultivars at harvest (day 0) and after 9 days at 1 °C. (**A**,**D**,**G**,**J**) January harvest; (**B**,**E**,**H**,**K**) February harvest; (**C**,**F**,**I**,**L**) March harvest. Bars are means of 3 biological replicates of 15 strawberries each. The letters above each bar denote significant differences (*p* < 0.05) between cultivars and days based on Tukey’s HSD test; bars with the same letter are not significantly different.

Sucrose, glucose, and fructose are the main sugars found in strawberries and, in general, sucrose contributes the least to the total sugar content in strawberries. In contrast, glucose and fructose concentrations are about a 1:1 ratio [44]. Some have suggested that the lower sucrose content in strawberry fruit may result from the hydrolysis of sucrose to glucose and fructose after translocation from the plant leaves [45]. In our study, sucrose content was very low in strawberries harvested in January but increased as the season progressed (Figure 6A–C). On the other hand, the glucose, fructose, and total sugar contents were lower in fruit harvested in March than in January, regardless of the cultivar (Figure 6D). Total sugar content of Pearl harvested in January was 89.95 g 100 g^−1^ DW. In contrast, in March, it declined to 67.80 g 100 g^−1^ DW. Similarly, the total sugar content of Brilliance harvested in January was 69.36 g 100 g^−1^ DW and declined to 50.30 g 100 g^−1^ DW in March. In a previous study, Wang and Camp [22] found that strawberry fruits from plants grown in cooler day/night temperatures have higher fructose, glucose, and total carbohydrates than those produced during warmer weather. Another study also showed that higher temperatures during the season contribute to a decline in the synthesis of sugars [23]. The difference between daytime and night temperatures (DIF) also affects the sugar content in strawberry fruit. For example, when the DIF is 12 °C, fructose, sucrose, and glucose contents accumulate in strawberry fruit, while increasing the DIF results in a decrease in all three sugars [21]. In addition, while Pearl and Brilliance’s respiration rate (RR) was not measured, it has been shown previously by Barrios et al. [20] that strawberries grown at higher temperatures have increased RR, which might have contributed to decreased total sugar content in Pearl and Brilliance as the temperatures raised during the season (Table 1). Wu et al. [21] also reported that strawberry RR increases during the day, while, at night, it decreases with an increase in DIF. The higher the night temperature, the higher the enzymatic activity and, consequently, the higher the RR. Thus, at low temperatures, the fruit RR will be lower, and the consumption of sugars will, therefore, also be lesser.

At harvest, sucrose content was significantly higher in Brilliance than in Pearl, particularly in fruit harvested in March (Figure 6C). Still, on average, Pearl had significantly lower sucrose levels than Brilliance (Table 2). On the other hand, at harvest, glucose, fructose, and total sugars were, in general, significantly higher in Pearl than in Brilliance (Table 2), but after storage, the difference lessened (Figure 6D–L). Hence, after 9 days of cold storage, sucrose content decreased significantly and, in some cases, was below detectable levels. Similar trends were observed for glucose, fructose, and total sugar content. A decrease in sugar content often results from increased metabolic activity after the fruit is detached from the mother plant. Molinett et al. [33] reported a sharp increase in the RR of white-fruited Chilean strawberries from harvest to after 6 days at 20 °C. The increase in the overall rate of metabolic reactions with increased storage time contributes to the breakdown of sugars, depleting fruit reserves [46].

#### 3.3.4. Ascorbic Acid Content

**Figure 7:**
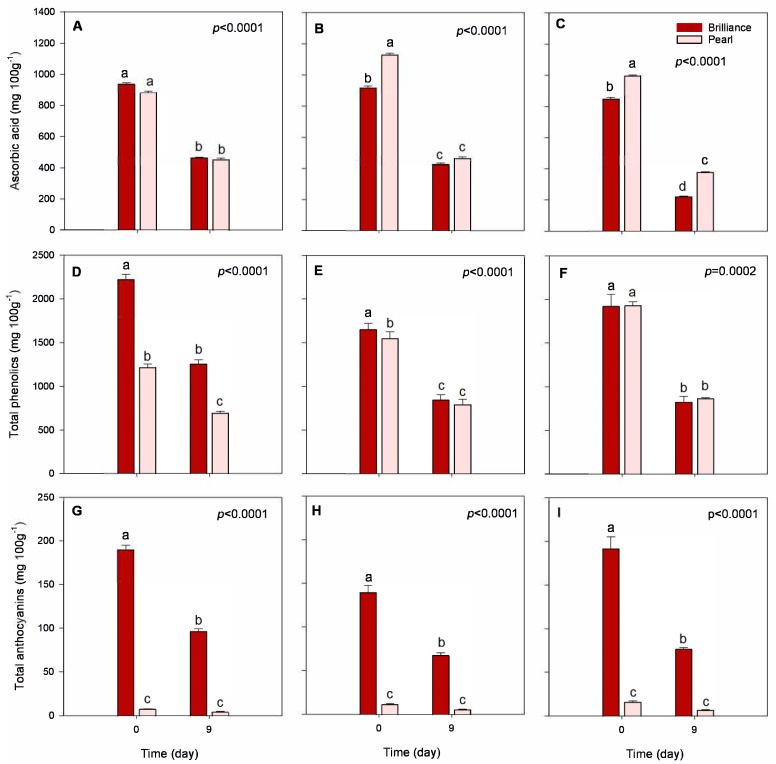
Total ascorbic acid, phenolics, and anthocyanin contents of Brilliance and Pearl strawberry cultivars at harvest (day 0) and after 9 days at 1 °C. (**A**,**D**,**G**) January harvest; (**B**,**E**,**H**) February harvest; (**C**,**F**,**I**) March harvest. Bars are means of 3 biological replicates of 15 strawberries each. The letters above each bar denote significant differences (*p* < 0.05) between cultivars and days based on Tukey’s HSD test; bars with the same letter are not significantly different.

Variability in ascorbic acid (AA) content during the season seemed to be cultivar-dependent. That is, Pearl strawberries harvested in January had lower AA contents than those harvested in March (879.90 and 995.26 mg 100 g^−1^ DW, respectively), but, on the other hand, Brilliance strawberries harvested in January had higher AA content than fruit harvested in March (939.50 and 848.78 mg 100 g^−1^ DW, respectively). Kim et al. [47] noted that the AA content of some strawberry cultivars was more clearly affected by changes in environmental conditions than others, which may explain the differences between Pearl and Brilliance tolerance to increased temperatures during the season. Some other studies have shown that higher temperatures during the season contribute to a decline in the synthesis of AA [22,23], and others reported lower AA contents in red-fruited strawberries grown at warmer temperatures [47]. Furthermore, Wang and Camp [22] noted that strawberries grown under shady conditions tend to have lower AA contents than those exposed to sunlight, suggesting that sunny days favor AA synthesis more than cloudy days. In addition to this, the difference between day and night temperatures also affects fruit metabolism. Higher daytime temperatures and stronger radiation favor the accumulation of AA [21]. Thus, in our study, the higher UV index measured in March (Table 1) might have contributed to the increase in AA observed in Pearl strawberries but not in Brilliance. On average, AA content measured at harvest (day 0) was higher in Pearl than in Brilliance (Table 2), particularly in fruit harvested later during the season (Figure 7A–C).

After cold storage, AA content significantly decreased, but—except for fruit harvested in March, where, compared to Brilliance, Pearl remained the cultivar with higher AA content after storage (221.85 versus 375.98 mg 100 g^−1^ DW, respectively)—there was no significant difference in the AA content between the two strawberry cultivars. However, Pearl tended to maintain slightly better AA levels during cold storage. That is, after 9 days, the decrease in AA content of Pearl strawberries was, depending on the season, between 48.6 and 62.2%, whereas that for Brilliance was between 50.5 and 73.9%.

#### 3.3.5. Total Phenolic and Anthocyanin Contents

It has been shown that there is a considerable difference in anthocyanin (ANC) and total phenolic contents (TPC) between different strawberry cultivars, as well as variability in response to UV light exposure in terms of polyphenol accumulation [10,48,49]. In our study, total phenolic content (TPC) was, on average, at harvest, higher in Brilliance than in Pearl (Table 2) and comparable to the values reported by Cheel et al. [50,51]. Red-fruited strawberry *Fragaria vesca* had a higher TPC (268.1 mg 100 g^−1^ FW) than the white strawberry *F. chiloensis* (106.3 mg 100 g^−1^ FW), while the TPC of the commercial cultivar ‘Chandler’ was approximately 150 mg 100 g^−1^ FW. Wang et al. [52] also measured lower TPC in the white-fruited strawberry cultivar ‘Baiuy’ than in red-fruited cultivars, suggesting that the differences are due to some polyphenols, such as anthocyanins, being lower in the white strawberry. Others have, however, reported different TPC values for white strawberries [15,53] compared to the average values measured in Pearl strawberries. Although TPC was significantly higher in Brilliance than in Pearl harvested in January (2221.14 and 1215.45 mg 100 g^−1^ DW, respectively) and February (1651.85 and 1544.70 mg 100 g^−1^ DW, respectively), there was no significant difference in TPC between the cultivars harvested in March (1920.21 and 1926.78 mg 100 g^−1^ DW, respectively) (Figure 7D–F). Thus, as the temperature and UV index increased during the season (Table 1), the differences in TPC between Brilliance and Pearl became smaller, likely because of the increase in Pearl’s red coloration. Palmieri et al. [10] reported that strawberry TPC varies depending on the cultivar, temperature, UV radiation, and sunshine duration. However, higher temperatures during the season generally contribute to a higher accumulation of strawberry TPC [54]. After storage, the TPC significantly decreased for both cultivars. Brilliance and Pearl harvested in January showed a 43.0% decrease in TPC after 9 days of storage (Figure 7D). For fruit harvested in February, both cultivars showed a 49.0% decrease from harvest to after storage (Figure 7E). Finally, Brilliance harvested in March showed a 57.0% decrease, whereas Pearl had a 53.0% decrease after 9 days of storage (Figure 7F). In a previous study, Saavedra et al. also reported a reduction in TPC of white-fruited strawberry *F. chiloensis* during storage for 72 h at 22 °C [41].

At harvest, total anthocyanin content (ANC) was, on average, approximately 14 times higher in Brilliance (11.91 mg 100 g^−1^ FW; 173.7 mg 100 g^−1^ DW) than in Pearl (0.83 mg 100 g^−1^ FW; 11.6 mg 100 g^−1^ DW) (Table 2, Figure 7G–I). Similarly, Simirgiotis et al. [16] reported that the ANC in ‘Chandler’ strawberries was 13 times higher than in the white-fruited *F. chiloensis* (27.9 and 2.20 100 g^−1^ FW, respectively). However, the amount of ANC measured in Pearl at harvest was much lower than the values previously reported by others [15,16,19,51], possibly due to cultivar and environmental differences. As the season progressed, there was an increase in the initial ANC of both strawberry cultivars, which agrees with the increased red coloration visually observed and the data obtained from analytical color measurements (Figure 1, Figure 2 and Figure 3). This was most likely due to the increased UV radiation the fruits were exposed to during growth and maturation (Table 1). Cominelli et al. [9] showed that light exposure is conducive to anthocyanin accumulation because some key regulators of flavonoid biosynthesis are light-induced. Kim et al. [23] also reported that when day/night temperatures increase, so do the anthocyanin contents of strawberry fruit. In a previous study, Noriega et al. [19] reported higher ANC values for *F. chiloensis* ranging from 1.59 to 7.86 mg 100 g^−1^ FW and attributed the unusually higher values to higher temperatures during the season. The increase in ANC seems to act like a visible light shield, attenuating the incoming light waves and protecting the fruit’s photosynthetic tissues and its organelles, such as the chloroplasts, from the damaging high temperatures and solar UV radiation [7,8,9].

Although there was an increase in ANC throughout the season, after storage, there was a consistent and significant decrease in ANC for both cultivars (Figure 7G–I). Yet, Brilliance strawberries had, on average, a significantly greater ANC after 9 days of storage (80.2 mg 100 g^−1^ DW) than Pearl (5.5 g mg^−1^ DW). Brilliance harvested in January showed a 49.0% decrease in ANC, while Pearl had a 41.0% decrease. Brilliance and Pearl were much closer regarding the percent decrease from harvest to after storage for the February and March harvests. Brilliance ANC decreased by 52.0 and 60.0%, respectively, and Pearl saw a reduction of 53.0 and 58.0%, respectively. Conversely, in a previous study, Saavedra et al. [41] reported no significant changes in the anthocyanin content of white-fruited strawberry *F. chiloensis* peel during storage for 72 h at 22 °C.

### 3.4. Polyphenol Profiles: Differences between Pearl and Brilliance, Seasonal Variability, and Impact of Storage

**Figure 8:**
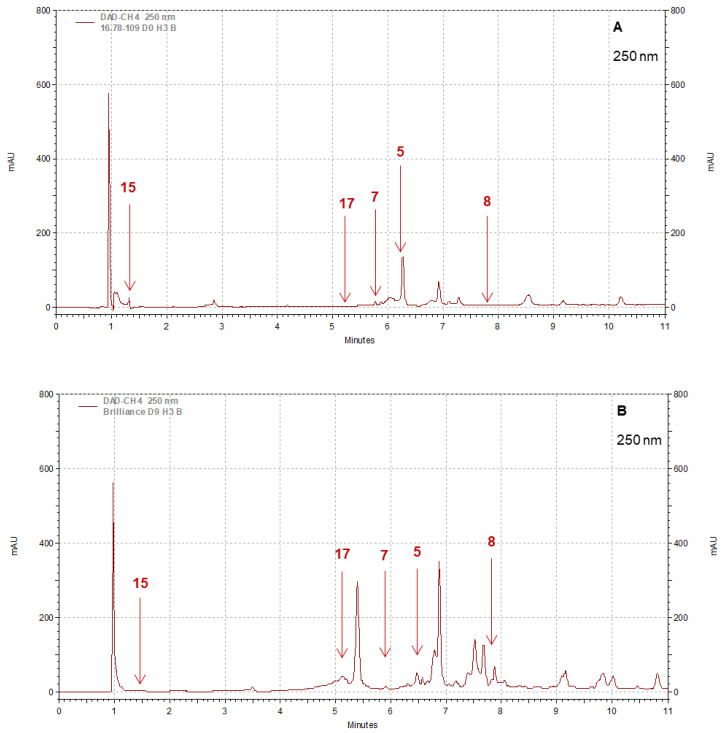
Example of HPLC chromatograms showing the polyphenols kaempferol 3-glucoside (5), quercetin 3-glucoside (7), quercetin (8), gallic acid (15), and ellagic acid (17) for Pearl (**A**) and Brilliance (**B**) strawberries harvested in March and measured after 9 days at 1 °C.

**Figure 9:**
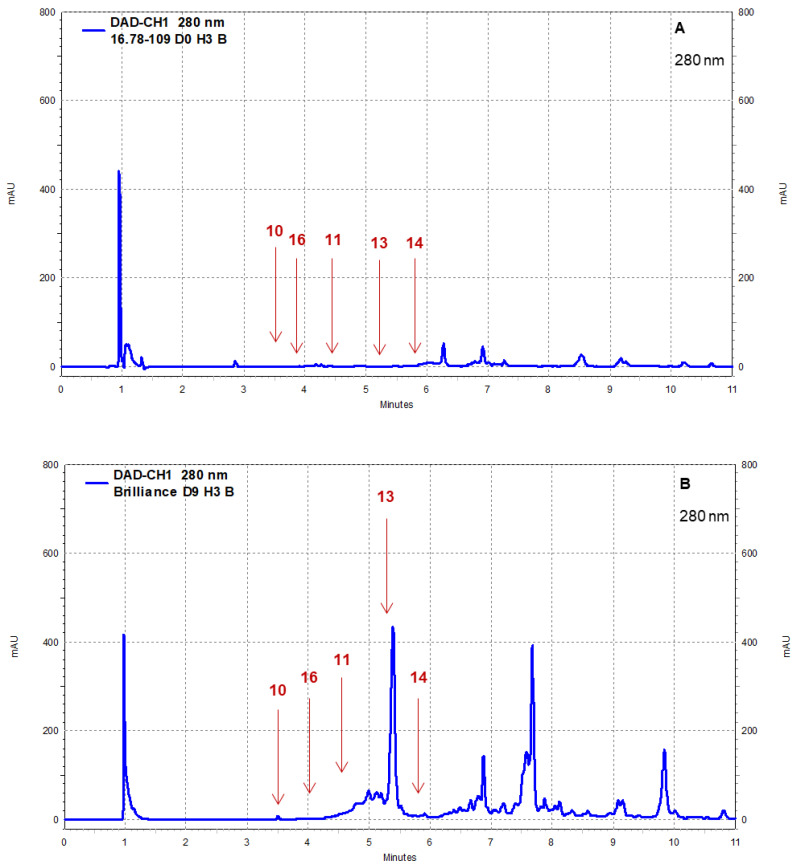
Example of HPLC chromatograms showing the polyphenols catechin (10), epicatechin (11), *p*-coumaric acid (13), ferulic acid (14), and chlorogenic acid (16) for Pearl (**A**) and Brilliance (**B**) strawberries harvested in March and measured after 9 days at 1 °C.

**Figure 10:**
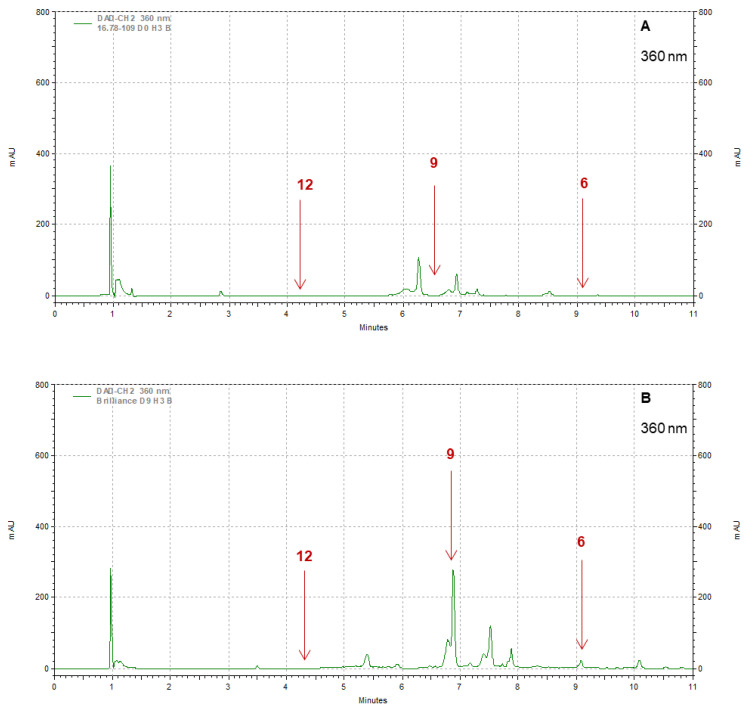
Example of HPLC chromatograms showing the polyphenols kaempferol (6), myricetin (9), and caffeic acid (12) for Pearl (**A**) and Brilliance (**B**) strawberries harvested in March and measured after 9 days at 1 °C.

**Figure 11:**
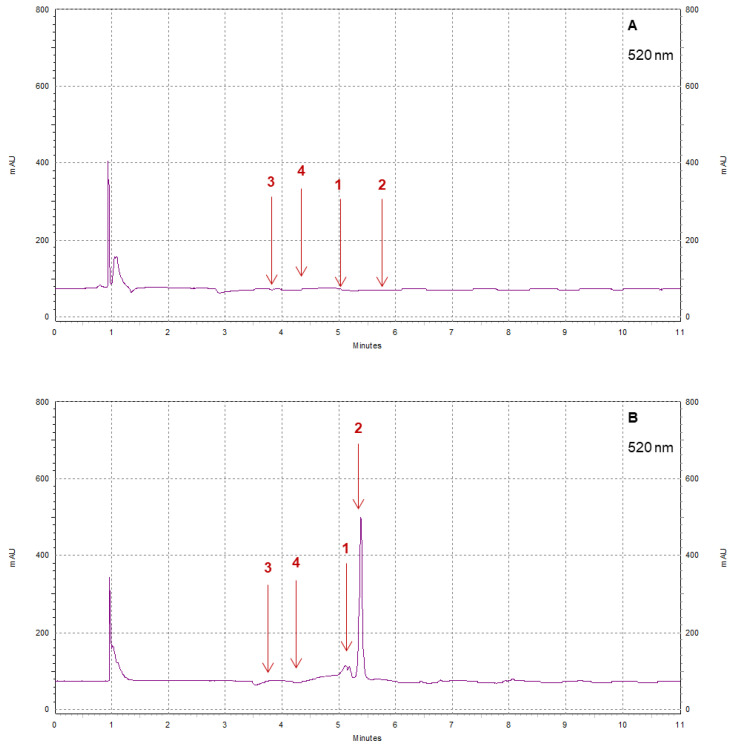
Example of HPLC chromatograms showing the polyphenols cyanidin (1), pelargonidin (2), cyanidin-3-glucoside (3), and pelargonidin 3-glucoside (4) for Pearl (**A**) and Brilliance (**B**) strawberries harvested in March and measured after 9 days at 1 °C.

Strawberry cultivars Pearl and Brilliance varied drastically from each other in terms of visual appearance and polyphenol profiles. As examples, the chromatograms shown in Figure 8, Figure 9, Figure 10 and Figure 11 indicate that the polyphenol profiles of these two strawberry cultivars are significantly different.

Pearl had, on average, at harvest, higher levels of kaempferol 3-glucoside, quercetin, quercetin 3-glucoside, and gallic acid but lower cyanidin, pelargonidin, pelargonidin 3-glucoside, epicatechin, and p-coumaric and ferulic acid contents than Brilliance (Table 2). The amounts of kaempferol, myricetin, catechin, chlorogenic acid, and ellagic acid were, on average, similar for Brilliance and Pearl, while caffeic acid was not detected in either cultivar. Previous studies showed that the red cultivar ‘Chandler’ had higher cyanidin 3-glucoside, pelargonidin 3-glucoside, and quercetin 3-glucoside levels and lower levels of ellagic acid but similar quercetin, kaempferol, kaempferol 3-glucoside, catechin, and epicatechin contents compared to white-fruited strawberries [16,55]. Surprisingly, no cyanidin 3-glucoside was detected in Pearl (Table 2). In contrast, others have reported that, in the white Chilean strawberry, cyanidin 3-glucoside and cyanidin derivatives are the major anthocyanin compounds present in the fruit, while ellagic acid and quercetin glucuronide are the main phenolic compounds [15,16,17,18,50,51]. Conversely, in our study, the amount of pelargonidin and pelargonidin 3-glucoside (5.21 mg 100 g^−1^ FW) represented the major pigments in Pearl instead of cyanidin and cyanidin 3-glucoside (0.82 mg 100 g^−1^ FW).

Compared to values obtained for Pearl, others have also reported higher values for cyanidin 3-glucoside and ellagic acid but similar or lower values for pelargonidin 3-glucoside in the white strawberry *F. chiloensis* [15,18,19]. Zhao et al. [56] reported very low levels of major anthocyanins in the white strawberry cultivar ‘Snow White’, but, like in our study, the concentrations of pelargonidin 3-glucoside were higher than that of cyanidin 3-glucoside. Previous studies also showed that the significant concentration of cyanidin-derived pigment in the white strawberry *F. chiloensis* is found in the achenes and not in the fruit because the achenes are the most colored part of the fruit [18,50,51]. Also, depending on the year, the levels of major anthocyanins in *F. chiloensis* ranged from 1.15 to 5.66 mg 100 g^−1^ FW for cyanidin 3-*O*-glucoside, while pelargonidin 3-*O*-glucoside contents varied between 0.10 to 0.43 mg 100 g^−1^ FW [3,15,16,17,18,50,51]. Accumulation of ellagic acid in the fruit also varied depending on the season (3.66 to 6.54 mg 100 g^−1^ FW) [19], the species, cultivar, and ripening stage of the strawberry fruit [49]. Thus, the differences between the white strawberry Pearl and other white-fruited strawberry cultivars regarding the major polyphenols, and particularly the types and amounts of anthocyanins present in the fruit, are most likely related to the coloration of the fruit at harvest but also influenced by the different growing conditions and genetic makeup. Although there is little accumulation of pelargonidin 3-glucoside in the white strawberry, it can increase when the receptacle has a slight pink color [13], which was the case for Pearl. In the white Chilean strawberry and white strawberry mutants, important enzymes involved in the flavonoid biosynthesis pathway are downregulated—namely, chalcone synthase, dihidroflavonol reductase, flavone 3-hydroxylase, and methyltransferase—thus, the production of several compounds, including pelargonidin, affecting the red color of strawberry is reduced [12,14,55].

Polyphenol profiles of Pearl and Brilliance also showed some variability depending on the harvest time (Table 3, Table 4 and Table 5). In January, Brilliance had at harvest higher levels of cyanidin, pelargonidin, cyanidin 3-glucoside, pelargonidin 3-glucoside, quercetin 3-glucoside, quercetin, epicatechin, and p-coumaric, ferulic, and gallic acids than Pearl, but significantly lower levels of kaempferol 3-glucoside (Table 3). In February, the trend was slightly different because the two cultivars had fewer significant differences in certain polyphenols (Table 4). For example, Brilliance had higher levels of pelargonidin, pelargonidin 3-glucoside, kaempferol, epicatechin, and *p*-coumaric acid than Pearl. In contrast, Pearl had significantly higher kaempferol 3-glucoside, quercetin 3-glucoside, and ferulic and gallic acids than Brilliance. Finally, in March, Brilliance had higher levels of pelargonidin, cyanidin 3-glucoside, quercetin 3-glucoside, quercetin, and *p*-coumaric and ellagic acids than Pearl (Table 5). On the other hand, Pearl had significantly higher kaempferol 3-glucoside, myricetin, and gallic acid than Brilliance. Overall, at harvest, Brilliance had consistently, across harvests, higher pelargonidin and *p*-coumaric acid levels than Pearl, whereas the white-fruited strawberries had consistently higher kaempferol 3-glucoside than Brilliance. Several studies have also shown significant differences in individual polyphenol profiles between cultivars, and that preharvest conditions may also contribute to the variability [10,22,23,47,57]. For example, pelargonidin 3-glucoside content was higher in red-fruited strawberries grown under cooler temperatures but higher UV radiation (15.6 to 59.3 mg 100 g^−1^ FW) than under warmer temperatures but lower UV radiation (8.4 to 39.4 mg 100 g^−1^ FW) [47]. Also, shading contributes to a decrease in anthocyanin production, with fruit grown in full light conditions having 15 to 16% higher anthocyanin content than fruit grown in partial shade [11]. Another study also showed that strawberry flavonols and ellagitannins increased when sunshine duration increased and decreased with increments in temperature and UV radiation [10]. Wang and Campos [22] reported that the ellagic acid content of red-fruited strawberries decreases as the field temperature increases (from approximately 2.4 to 1.5 mg g^−1^ DW) and suggested that exposure to high temperatures may lead to a decrease in the synthesis of ellagic acid or increased degradation.

After cold storage, depending on the cultivar, the levels of most polyphenol compounds decreased while others tended to remain the same or increased slightly (Table 3, Table 4 and Table 5). For example, there was a decrease in all polyphenols for Pearl harvested in January, whereas, in Brilliance, cyanidin 3-glucoside, epicatechin, and chlorogenic acid contents significantly increased (Table 3). Compared to Pearl, Brilliance had, after storage, higher levels of most polyphenols, including cyanidin, cyanidin 3-glucoside, pelargonidin 3-glucoside, kaempferol, quercetin, myricetin, epicatechin, and *p*-coumaric, ferulic, gallic, chlorogenic, and ellagic acids. In the February harvest, there was an increase in pelargonidin, quercetin 3-glucoside, quercetin, and *p*-coumaric and ferulic acids in Brilliance (Table 4). In Pearl, all polyphenol compounds except quercetin and *p*-coumaric acid decreased after cold storage. Compared to Pearl, Brilliance had, after storage, higher levels of pelargonidin, quercetin 3-glucoside, quercetin, and *p*-coumaric and ferulic acids, whereas Pearl had, after storage, higher levels of chlorogenic acid than Brilliance (Table 4). In the March harvest, the only polyphenols that increased in Brilliance were kaempferol 3-glucoside and ferulic acid. In contrast, in Pearl, there was an increase in cyanidin 3-glucoside, myricetin, and epicatechin after cold storage (Table 5). Also, compared to Pearl, the cultivar Brilliance had higher levels of cyanidin, pelargonidin 3-glucoside, quercetin, and *p*-coumaric, ferulic, and ellagic acids but lower cyanidin 3-glucoside, kaempferol 3-glucoside, quercetin 3-glucoside, myricetin, and epicatechin. Overall, after cold storage, Brilliance had higher levels of quercetin and *p*-coumaric and ferulic acids than Pearl across all harvests, while the levels of other polyphenols varied depending on the harvest.

**Figure 12:**
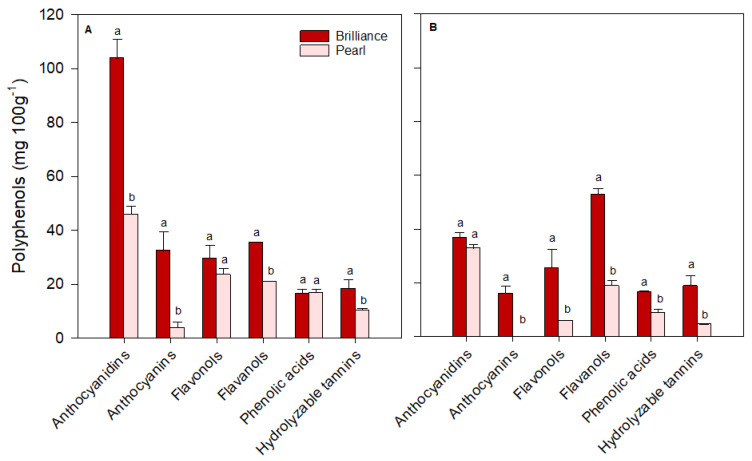
The concentration (mg 100 g^−1^ of fruit dry weight) of the major polyphenol groups in Brilliance and Pearl strawberry cultivars at harvest ((**A**); day 0) and after 9 days (**B**) at 1 °C (January harvest). The letters above each bar denote significant differences (*p* < 0.05) between cultivars based on Tukey’s HSD test; bars with the same letter are not significantly different.

**Figure 13:**
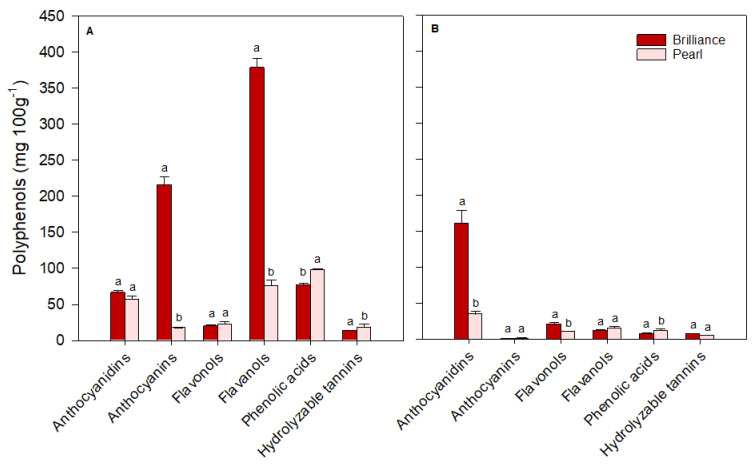
The concentration (mg 100 g^−1^ of fruit dry weight) of the major polyphenol groups in Brilliance and Pearl strawberry cultivars at harvest ((**A**); day 0) and after 9 days (**B**) at 1 °C (February harvest). The letters above each bar denote significant differences (*p* < 0.05) between cultivars and days based on Tukey’s HSD test; bars with the same letter are not significantly different.

**Figure 14:**
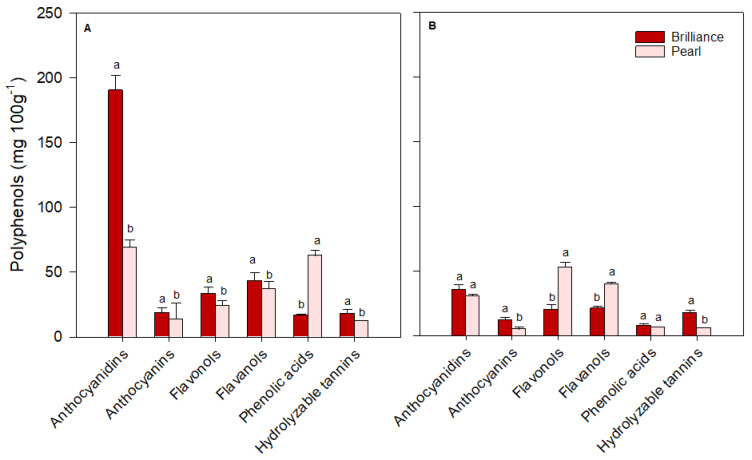
The concentration (mg 100 g^−1^ of fruit dry weight) of the major polyphenol groups in Brilliance and Pearl strawberry cultivars at harvest ((**A**); day 0) and after 9 days (**B**) at 1 °C (March harvest). The letters above each bar denote significant differences (*p* < 0.05) between cultivars and days based on Tukey’s HSD test; bars with the same letter are not significantly different.

For ease of comparison, polyphenols identified in strawberries were grouped into major classes: anthocyanidins (cyanidin plus pelargonidin), anthocyanins (cyanidin 3-glucoside plus pelargonidin 3-glucoside), flavonols (quercetin, kaempferol, quercetin 3-glucoside, kaempferol 3-glucoside, plus myricetin), flavanols (catechin plus epicatechin), phenolic acids (p-coumaric acid, ferulic acid, caffeic acid, gallic acid, plus chlorogenic acid) and hydrolyzable tannins (ellagic acid). There was some variability in the levels of each polyphenol group depending on the cultivar and harvest time. On the day of harvest (day 0), Brilliance had higher (January and March harvests) or similar (February harvest) levels of anthocyanidins than Pearl (Figure 12, Figure 13 and Figure 14). However, on average, Brilliance had twice the levels of anthocyanidins than Pearl (120.27 and 57.34 mg 100 g^−1^ DW). After storage, the levels of anthocyanidins significantly decreased in Brilliance, except for the February harvest, where there was an unexpected increase. After storage, the decrease in Pearl’s anthocyanidin contents was less marked than in Brilliance, except in the March harvest, where the reduction from initial values was significantly higher than in the other harvests (Figure 14). Consequently, the reduction in anthocyanidin content of Pearl was, after storage, slightly higher than in Brilliance. On average, storage for 9 days at 1 °C resulted in a 35.4% and 42.9% reduction in the anthocyanidin contents of Brilliance and Pearl strawberries, respectively.

As expected, anthocyanin levels were significantly higher in Brilliance than in Pearl, particularly in the two first harvests (32.42 and 215.63 mg 100 g^−1^ DW for Brilliance and 3.81 and 17.66 mg 100 g^−1^ DW for Pearl, respectively) (Figure 12 and Figure 13). On average, anthocyanin content in Brilliance was 89.02 mg 100 g^−1^ DW, while in Pearl it was 11.84 mg 100 g^−1^ DW. The higher levels of anthocyanins in Brilliance than in Pearl were expected since pelargonidin 3-glucoside is the primary determinant of a strawberry’s red color. The less marked difference in anthocyanin content between the white and red cultivars in the March harvest (19.00 and 14.06 mg 100 g^−1^, respectively) was most likely due to increased red coloration in the white strawberries as the season progressed (Figure 1). After cold storage, the decrease was substantially higher in Brilliance than in Pearl, particularly in the February harvest (Figure 13). Although the levels of anthocyanins were much high in Brilliance than in Pearl at harvest, the decline for both cultivars was dramatic after cold storage. On average, storage for 9 days at 1 °C resulted in an 89.2% and 82.2% reduction in the anthocyanin content of Brilliance and Pearl strawberries, respectively.

For the flavonols, there was no significant difference between Brilliance and Pearl in the two first harvests (29.56 and 20.30 mg 100 g^−1^ DW; 23.57 and 22.72 mg 100 g^−1^ DW, respectively) (Figure 12 and Figure 13). In March, Brilliance had a slightly higher content of flavonols than Pearl (33.64 and 24.07 mg 100 g^−1^ DW, respectively) (Figure 14). On average, at harvest, the flavonol content of Brilliance was 27.83 mg 100 g^−1^ DW whereas Pearl flavonol content was slightly lower, 23.45 mg 100 g^−1^. After storage, there was a decrease in flavonol content for both cultivars, except in the March harvest, where the flavonol content of Pearl increased slightly. Regardless, the decline in flavonol content was more dramatic in Brilliance than in Pearl. On average, storage for 9 days at 1 °C resulted in a 19.6% and 1.2% reduction in the flavonol content of Brilliance and Pearl strawberries, respectively.

During the season, there was significant variability in the levels of flavanols measured at harvest for both Brilliance and Pearl (Figure 12, Figure 13 and Figure 14). The levels of flavanols in Brilliance were notably higher in February (378.39 mg 100 g^−1^ DW), more than 8 times higher than in the January and March harvests (35.51 and 43.49 mg 100 g^−1^ DW, respectively). Although Brilliance had, on average, higher concentrations of flavanols than Pearl (152.46 and 44.79 mg 100 g^−1^ DW, respectively), the trend during the season was similar for both cultivars. Thus, the flavanol content of Pearl was significantly higher in February (76.06 mg 100 g^−1^ DW) than in January or March (21.04 and 37.28 mg 100 g^−1^ DW, respectively). After storage, Brilliance’s flavanol concentration significantly decreased in the two last harvests (Figure 13 and Figure 14). However, there was an increase in flavanol concentration after storage in the fruit harvested in January (Figure 12). On the other hand, after storage, flavanol content in Pearl decreased in fruit harvested in January and February but slightly increased in March. Nonetheless, the decrease in phenolic acid was more dramatic in Brilliance than in Pearl. Therefore, on average, storage for 9 days at 1 °C resulted in an 81.2% and 45.05% reduction in the flavanol content of Brilliance and Pearl strawberries, respectively.

For the phenolic acids, there was no significant difference between Brilliance and Pearl harvested in January (16.65 and 16.83 mg 100 g^−1^ DW, respectively) (Figure 12). However, in February and particularly in March, phenolic acid levels at harvest were significantly higher in Pearl than in Brilliance (97.61 and 62.80 mg 100 g^−1^ DW; 76.82 and 16.94 mg 100 g^−1^ DW, respectively) (Figure 13 and Figure 14). On average, phenolic acids were at harvest higher in Pearl (59.08 mg 100 g^−1^ FW) than in Brilliance (36.80 mg 100 g^−1^ DW). After cold storage, the levels of phenolic acids significantly decreased for both cultivars, particularly in the last two harvests. Therefore, on average, storage for 9 days at 1 °C resulted in a 70.7% and 84.3% reduction in the flavanol content of Brilliance and Pearl strawberries, respectively.

Differences in hydrolyzable tannins (ellagic acid) between Brilliance and Pearl were less significant at harvest than for the other polyphenol groups. Yet, they were slightly higher in Brilliance than in Pearl harvested in January and March (18.46 and 18.04 mg 100 g^−1^ DW; 10.42 and 12.73 mg 100 g^−1^ DW, respectively) (Figure 12 and Figure 14), whereas the opposite was observed in the February harvest, where Pearl had a higher content of hydrolyzable tannins than Brilliance (18.57 and 13.64 mg 100 g^−1^ DW) (Figure 13). Regardless of the difference between harvests, on average, Brilliance had a higher content of hydrolyzable tannins than Pearl (1.15 and 1.03 mg 100 g^−1^ FW or 16.71 and 13.91 mg 100 g^−1^ DW, respectively). In a previous study, Zhao et al. [58] reported lower ellagic acid contents (approximately 0.45 mg 100 g^−1^ FW) for the white strawberry cultivar ‘Snow White’. However, they also suggested that the difference in the amount of ellagic acid between laboratories might be related to the different analytical procedures used [58]. After storage, there was a decrease in the levels of ellagic acid in Pearl, while in Brilliance the levels remained similar after storage, particularly in fruit from January and March harvests. Nonetheless, the decrease in the hydrolyzable tannins was more dramatic in Pearl than in Brilliance. Therefore, on average, storage for 9 days at 1 °C resulted in a 12.4% and 61.0% reduction in the flavanol content of Brilliance and Pearl strawberries, respectively.

## 4. Conclusions

This study showed that the white-fruited strawberry Pearl has different physicochemical characteristics and a different polyphenolic profile than the commercial strawberry cultivar Brilliance. Although the harvest date contributed to significant differences in the quality of the fruit, Pearl was, on average, softer and had lower total phenolic and anthocyanin contents but was less acidic and had higher total sugars and ascorbic acid contents than Brilliance. Pearl’s major polyphenols were quercetin 3-glucoside, kaempferol 3-glucoside, quercetin, and gallic acid, while for Brilliance epicatechin, pelargonidin, pelargonidin 3-glucoside, and ferulic acid were the major polyphenol compounds identified. Pearl seems highly sensitive to high field temperatures and UV radiation, which seem to trigger anthocyanin synthesis as a protective response. The increase in total anthocyanin contents, particularly in the third harvest when the field temperatures and UV radiation were higher, resulted in fruit with a noticeable pink blush and not creamy white. Thus, the plant and fruit would benefit from UV protection during growth if commercialized as a white or pearl strawberry fruit. After cold storage, Pearl lost, on average, less weight than Brilliance and showed a less dramatic decline in individual polyphenols. Pearl and Brilliance anthocyanins and phenolic acids were the groups most affected by cold storage because they showed the highest reduction from harvest to the end of storage. Cold storage (1 °C) also had different consequences on other polyphenols, but the result was cultivar-dependent. Brilliance’s flavanols were more affected by storage than ellagic acid, whereas Pearl’s ellagic acid was more affected by storage than flavonols. Overall, white-fruited strawberries have a unique appearance and, compared to red-fruited strawberries, they are sweeter, have an excellent bioactive profile, and can maintain a good quality profile during cold storage.

## Figures and Tables

**Table 1:** Weather conditions at harvest in the 2021 strawberry season ^a^.

Harvest Date	Temperature (°C)			Rainfall (mm)	Daily UV Index (Max AVG)
		Max	Min			AVG
18 January	17.8	6.1	12.5	0	4
10 February	27.2	18.9	21.1	0	5
1 March	28.3	21.7	24.3	0	6

Max = maximum; Min = minimum; AVG = average. ^a^ Source: Florida Automated Weather Network (https://fawn.ifas.ufl.edu/data/reports (accessed on 12 July 2021)).

**Table 2:** Chemical compounds and major polyphenols measured at harvest (day 0) in Brilliance and Pearl strawberry cultivars expressed on a fresh and dry weight basis ^a^.

Compound	Brilliance		Pearl	
	Mean FW ± SE	Mean DW ± SE	Mean FW ± SE	Mean DW ± SE
Acidity (%)	0.78 ± 0.04	11.36 ± 0.40	0.65 ± 0.06	8.39 ± 0.33
SSC (%)	7.17 ± 0.22	95.74 ± 2.13	7.97 ± 0.53	96.47 ± 1.82
Sugars (g 100 g^−1^)				
Sucrose	0.26 ± 0.05	3.92 ± 1.10	0.04 ± 0.01	0.64 ± 0.21
Glucose	2.03 ± 0.11	29.45 ± 2.15	2.67 ± 0.17	34.19 ± 1.33
Fructose	2.10 ± 0.14	30.44 ± 2.67	2.96 ± 0.18	38.18 ± 1.28
Total Sugars	4.39 ± 0.20	63.81 ± 3.87	5.67 ± 0.34	73.02 ± 2.45
Bioactive compounds (mg 100 g^−1^)				
Total ascorbic acid	61.78 ± 1.54	900.53 ± 21.69	74.63 ± 2.27	1015.83 ± 40.25
Total phenolics	132.97 ± 4.49	1931.06 ± 95.10	114.51 ± 1.83	1605.66 ± 110.33
Total anthocyanins	11.91 ± 0.44	173.67 ± 9.77	0.83 ± 0.03	12.10 ± 1.37
Polyphenols (mg 100 g^−1^)				
Cyanidin	0.98 ± 0.09	14.34 ± 1.17	0.82 ± 0.02	11.46 ± 0.75
Pelargonidin	7.18 ± 1.24	105.94 ± 19.37	3.40 ± 0.18	47.32 ± 3.73
Cyanidin-3-glucoside	0.12 ± 0.02	1.72 ± 0.51	0.00 ± 0.00	0.00 ± 0.00
Pelargonidin 3-glucoside	5.13 ± 2.45	76.11 ± 36.30	1.81 ± 0.33	21.85 ± 3.08
Kaempferol 3-glucoside	1.27 ± 0.17	17.80 ± 2.52	7.32 ± 0.28	102.21 ± 6.91
Kaempferol	1.06 ± 0.09	15.61 ± 1.34	0.93 ± 0.04	12.66 ± 0.55
Quercetin	3.51 ± 0.19	7.74 ± 2.99	4.62 ± 0.02	0.87 ± 0.27
Quercetin 3-glucoside	0.52 ± 0.31	41.39 ± 4.46	7.86 ± 2.23	86.22 ± 31.76
Myricetin	1.39 ± 0.14	20.10 ± 1.79	1.70 ± 0.16	22.58 ± 1.85
Catechin	2.01 ± 0.59	29.63 ± 8.82	2.03 ± 0.67	30.43 ± 9.53
Epicatechin	8.31 ± 3.46	122.83 ± 51.23	1.19 ± 0.17	17.33 ± 2.88
Caffeic acid	0.00 ± 0.00	0.00 ± 0.00	0.00 ± 0.00	0.00 ± 0.00
*p*-Coumaric acid	1.16 ± 0.14	15.65 ± 1.89	0.04 ± 0.02	0.66 ± 0.32
Ferulic acid	3.60 ± 0.48	53.28 ± 6.25	1.82 ± 0.60	25.89 ± 8.54
Gallic acid	2.32 ± 0.70	34.09 ± 10.33	3.90 ± 0.78	60.89 ± 11.52
Chlorogenic acid	0.19 ± 0.00	2.71 ± 0.04	0.24 ± 0.04	3.47 ± 0.65
Ellagic acid	1.15 ± 0.11	16.71 ± 1.51	1.03 ± 0.13	14.34 ± 1.96

^a^ Means are averages of three harvests per cultivar; *n* = 3 harvests × 3 replicates of 15 strawberries each. FW = fresh weight, DW = dry weight, SE = standard error.

**Table 3:** The concentration (mg 100 g^−1^ of fruit dry weight ± SE) of the major polyphenols measured in Brilliance and Pearl strawberry cultivars at harvest (day 0) and after 9 days at 1 °C (January harvest) ^a^.

Polyphenols	Day 0		Day 9	
	Brilliance	Pearl	Brilliance	Pearl
Anthocyanidins				
Cyanidin	15.77 ± 3.52 a	8.77 ± 0.10 b	12.16 ± 1.66 a	4.85 ± 0.13 b
Pelargonidin	88.11 ± 5.12 a	37.09 ± 3.50 b	24.68 ± 2.98 a	28.07 ± 1.18 a
Anthocyanins				
Cyanidin 3-glucoside	3.81 ± 2.85 a	0.00 ± 0.00 b	5.20 ± 2.60 a	0.00 ± 0.00 b
Pelargonidin 3-glucoside	32.42 ± 0.29 a	0.00 ± 0.00 b	10.11 ± 0.20 a	0.00 ± 0.00 b
Flavonols				
Kaempferol 3-glucoside	16.01 ± 2.49 b	79.105 ± 9.79 a	12.73 ± 1.60 b	24.75 ± 1.63 a
Kaempferol	12.31 ± 0.28 a	10.81 ± 1.34 a	11.59 ± 0.16 a	5.18 ± 0.22 b
Quercetin 3-glucoside	41.06 ± 2.91 a	16.78 ± 5.85 b	9.25 ± 0.14 a	5.01 ± 1.06 a
Quercetin	5.21 ± 1.44 a	0.82 ± 0.81 b	4.29 ± 0.32 a	0.23 ± 0.02 b
Myricetin	24.35 ± 2.40 a	22.76 ± 2.10 a	21.35 ± 7.22 a	5.84 ± 0.07 b
Flavanols				
Catechin	13.29 ± 0.01 a	10.46 ± 0.02 a	6.76 ± 0.12 a	8.18 ± 0.59 a
Epicatechin	22.23 ± 0.09 a	10.58 ± 0.16 b	46.25 ± 2.16 a	10.73 ±1.47 b
Phenolic acids				
Caffeic acid	0.00 ± 0.00 a	0.00 ± 0.00 a	0.00 ± 0.00 a	0.00 ± 0.00 a
p-Coumaric acid	22.18 ± 0.91 a	0.00 ± 0.00 b	26.39 ± 4.60 a	0.00 ± 0.00 b
Ferulic acid	73.11 ± 4.23 a	13.96 ± 1.94 b	7.92 ± 0.10 a	3.85 ± 0.54 b
Gallic acid	14.10 ± 0.13 a	14.82 ± 1.68 a	6.42 ± 0.22 a	5.50 ± 0.08 b
Chlorogenic acid	2.55 ± 0.01 a	2.01 ± 0.01 a	10.26 ± 0.22 a	3.38 ± 1.39 b
Hydrolyzable tannins				
Ellagic acid	18.46 ± 4.47 a	10.42 ± 0.74 b	18.95 ± 3.69 a	4.65 ± 0.05 b

^a^ Letters after averages (*n* = 3 replicates of 15 strawberries each) denote significant differences (*p* < 0.05) between treatments based on Tukey’s HSD test; averages followed by the same letter are not significantly different. SE = standard error.

**Table 4:** The concentration (mg 100 g^−1^ of fruit dry weight ± SE) of the major polyphenols measured in Brilliance and Pearl strawberry cultivars at harvest (day 0) and after 9 days at 1 °C (February harvest) ^a^.

Polyphenols	Day 0		Day 9	
	Brilliance	Pearl	Brilliance	Pearl
Anthocyanidins				
Cyanidin	11.83 ± 0.01 a	11.44 ± 0.13 a	8.15 ± 1.25 a	6.76 ± 0.57 a
Pelargonidin	54.55 ± 4.23 a	45.54 ± 5.97 b	152.30 ± 18.48 a	27.89 ± 4.31 a
Anthocyanins				
Cyanidin 3-glucoside	0.00 ± 0.00 a	0.00 ± 0.00 a	0.00 ± 0.00 a	0.00 ± 0.00 a
Pelargonidin 3-glucoside	215.63 ± 15.54 a	17.66 ± 0.23 b	0.62 ± 0.02 a	1.22 ± 0.71 a
Flavonols				
Kaempferol 3-glucoside	23.07 ± 4.24 b	100.15 ± 8.43 a	6.72 ± 0.69 a	5.00 ± 0.86 a
Kaempferol	20.44 ± 1.06 a	13.48 ± 0.19 b	7.92 ± 0.94 a	6.32 ± 0.45 a
Quercetin 3-glucoside	50.56 ± 4.70 b	194.35 ± 0.38 a	81.98 ± 9.62 a	4.42 ± 0.84 b
Quercetin	0.56 ± 0.06 a	0.83 ± 0.28 a	13.24 ± 1.89 a	4.18 ± 1.01 b
Myricetin	19.75 ± 1.58 a	21.90 ± 0.36 a	7.94 ± 0.75 a	6.59 ± 0.32 a
Flavanols				
Catechin	60.90 ± 10.91 a	59.04 ± 9.70 a	6.08 ± 0.23 a	8.61 ± 1.88 a
Epicatechin	317.50 ± 33.58 a	17.02 ± 3.02 b	5.69 ± 1.52 a	6.49 ± 1.01 a
Phenolic acids				
Caffeic acid	0.00 ± 0.00 a	0.00 ± 0.00 a	0.00 ± 0.00 a	0.00 ± 0.00 a
p-Coumaric acid	10.62 ± 3.27 a	1.30 ± 0.55 b	16.0 ± 8.34 a	2.47 ± 0.40 b
Ferulic acid	37.58 ± 6.01 b	53.53 ± 10.62 a	48.99 ± 5.71 a	4.85 ± 0.51 b
Gallic acid	74.10 ± 0.93 b	92.61 ± 2.37 a	6.68 ± 0.72 a	7.42 ± 1.10 a
Chlorogenic acid	2.74 ± 0.01 a	4.99 ± 1.00 a	1.17 ± 0.04 b	4.85 ± 0.84 a
Hydrolyzable tannins				
Ellagic acid	13.64 ± 0.27 a	18.57 ± 5.38 a	7.24 ± 0.89 a	5.44 ± 0.06 a

^a^ Letters after averages (*n* = 3 replicates of 15 strawberries each) denote significant differences (*p* < 0.05) between treatments based on Tukey’s HSD test; averages followed by the same letter are not significantly different. SE = standard error.

**Table 5:** The concentration (mg 100 g^−1^ of fruit dry weight ± SE) of the major polyphenols measured in Brilliance and Pearl strawberry cultivars at harvest (day 0) and after 9 days at 1 °C (March harvest) ^a^.

Polyphenols	Day 0		Day 9	
	Brilliance	Pearl	Brilliance	Pearl
Anthocyanidins				
Cyanidin	15.4 ± 1.37 a	13.26 ± 1.44 a	14.28 ± 5.08 a	6.46 ± 0.61 b
Pelargonidin	175.15 ± 7.34 a	55.91 ± 3.66 b	21.42 ± 3.18 a	24.11 ± 3.33 a
Anthocyanins				
Cyanidin 3-glucoside	1.36 ± 0.66 a	0.01 ± 0.00 b	1.19 ± 0.19 b	3.42 ± 0.76 a
Pelargonidin 3-glucoside	12.70 ± 0.56 a	18.99 ± 2.58 a	10.93 ± 0.93 a	1.68 ± 0.99 b
Flavonols				
Kaempferol 3-glucoside	14.33 ± 1.33 b	119.68 ± 4.78 a	20.22 ± 3.58 b	54.27 ± 1.54 a
Kaempferol	14.08 ± 0.20 a	13.08 ± 0.03 a	7.40 ± 1.68 a	5.65 ± 0.42 a
Quercetin 3-glucoside	32.54 ± 11.60 a	24.37 ± 5.32 b	5.84 ± 1.18 b	9.85 ± 1.92 a
Quercetin	17.44 ± 3.81 a	0.93 ± 0.36 b	14.05 ± 3.61 a	2.82 ± 0.02 b
Myricetin	16.20 ± 1.49 b	23.14 ± 1.34 a	6.23 ± 0.18 b	49.87 ± 3.28 a
Flavanols				
Catechin	14.71 ± 0.14 a	15.14 ± 2.52 a	6.28 ± 0.93 a	8.59 ± 0.01 a
Epicatechin	28.78 ± 6.39 a	22.14 ± 7.28 a	15.02 ± 0.06 b	31.22 ± 9.48 a
Phenolic acids				
Caffeic acid	0.00 ± 0.00 a	0.00 ± 0.00 a	0.00 ± 0.00 a	0.00 ± 0.00 a
p-Coumaric acid	14.16 ± 1.80 a	0.46 ± 0.01 b	15.56 ± 1.40 a	0.57 ± 0.14 b
Ferulic acid	49.16 ± 4.73 a	6.22 ± 0.94 b	74.06 ± 11.33 a	5.96 ± 1.05 b
Gallic acid	14.11 ± 0.55 b	59.89 ± 5.46 a	5.05 ± 0.15 a	5.53 ± 0.13 a
Chlorogenic acid	2.83 ± 0.02 a	2.91 ± 0.01 a	2.80 ± 1.14 a	1.14 ± 0.03 a
Hydrolyzable tannins				
Ellagic acid	18.04 ± 2.36 a	12.73 ± 0.25 a	17.73 ± 1.82 a	6.20 ± 0.17 b

^a^ Letters after averages (*n* = 3 replicates of 15 strawberries each) denote significant differences (*p* < 0.05) between treatments based on Tukey’s HSD test; averages followed by the same letter are not significantly different. SE = standard error.

## Data Availability

The data presented in this study are available on request from the corresponding author.
